# Sex Differences in the In-Hospital Mortality of Patients with Acute Myocardial Infarction: A Cross-Sectional Study in 36 Hospitals Across Germany

**DOI:** 10.3390/medicina61050891

**Published:** 2025-05-14

**Authors:** Karel Kostev, Nimran Kaur, Sabine Kluge, Marcel Konrad, Jamschid Sedighi, Mark Lüdde

**Affiliations:** 1 IQVIA, Epidemiology, 60549 Frankfurt am Main, Germany; 2 University Hospital, Philipps University of Marburg, 35037 Marburg, Germany; 3 IQVIA, Epidemiology, Bangalore 560 103, India; nimran.kaur@iqvia.com; 4 IQVIA, Communications, 60549 Frankfurt am Main, Germany; sabine.kluge@iqvia.com; 5 Health & Social, FOM University of Applied Sciences for Economics and Management, 60486 Frankfurt am Main, Germany; konrad.marcel@web.de; 6 Medical Clinic I, Cardiology and Angiology, Justus-Liebig-University, 35292 Giessen, Germany; jamschid.sedighi@gmail.com (J.S.); mark.luedde@web.de (M.L.); 7 University Hospital, Christian-Albrechts-University of Kiel, 24118 Kiel, Germany

**Keywords:** myocardial infarction, sex differences, Germany, in-hospital mortality

## Abstract

*Background and Objectives:* Acute myocardial infarction (AMI) is one of the leading causes of mortality worldwide and caused ~1.8 million deaths in the European Union from 2012 to 2020. This study aimed to analyze and quantify sex-based disparities, identifying both clinical and systemic contributors to in-hospital mortality differences between male and female patients. *Materials and Methods:* This multicenter cross-sectional study from 36 hospitals across Germany included all hospitalized patients aged ≥18 years with admissions between January 2019 and December 2023 and a primary diagnosis of AMI. The primary outcome of the study was the prevalence of in-hospital mortality as a function of sex. Multivariable logistic regression analyses were conducted to assess the associations between female sex as compared with male sex and in-hospital mortality. *Results:* The present study included 9142 male and 4128 female patients with AMI. Women were significantly older than men (74.4 years versus 67.7 years). The proportion of non-ST elevation (NSTE-MI)-MI was higher in women than in men (70.7% versus 66.7%). Overall, in-hospital mortality was higher in women than in men (8.5% versus 7.1%). In a multivariable regression model, female sex was not significantly associated with in-hospital mortality (OR: 0.89; 95% CI: 0.77–1.04) irrespective of the MI type. *Conclusions:* There were no significant sex-based differences observed in the in-hospital mortality among patients suffering from AMI in Germany.

## 1. Introduction

Acute myocardial infarction (AMI) is one of the leading causes of mortality worldwide and the gravest clinical presentation of coronary artery disease. AMI affects nearly 3 million people globally, resulting in over 1 million deaths each year in the United States alone [1]. The number of deaths due to AMI in the European Union from 2012 to 2020 has been estimated at nearly 1.8 million (1 million men and 0.8 million women) [2].

Significant research has shown that sex-based differences affect both the presentation and outcomes of AMI. However, our understanding of the extent and implications of these disparities remains incomplete. Most studies on this topic state that men have a higher AMI incidence rate than women [3] and that women are less likely to undergo any treatment for the condition irrespective of their age [4]. Further evidence suggests that female patients experience higher in-hospital mortality rates than males, even after accounting for potential confounding factors [5]. Accordingly, the 30-day mortality risk following MI is twice as high in women as in men after adjustment for confounding variables in different countries [6].

The negative impact of female sex on post-MI outcomes also had an age-specific aspect, with younger and middle-aged women in particular faring worst regarding most outcomes [7]. Contradictorily, in 2017, about 70% of patients hospitalized in Germany for acute cardiac manifestations were men [8], though a decline of 2.6% per year in the age-standardized mortality of German men aged 25–74 years suffering from MI was noticed between 2004 and 2015 [9]. Although the short-term trends in AMI in-hospital case fatalities in Europe were published a decade ago [10], they were not stratified by sex or age group. As a result, chronic heart disease rates and long-term trends in in-hospital case-fatality rates [10] in Europe are impacted by a lack of understanding with regard to sex- and age-based differences in in-hospital mortality among AMI patients. This in turn affects the development of targeted interventions and appropriate clinical practice guidelines and impedes the optimization of outcomes.

Against this background, the aim of this study was to analyze differences in in-hospital mortality between male and female AMI patients in 36 hospitals throughout Germany.

## 2. Methods

### 2.1. Data Source

This multicenter cross-sectional study was based on data from the hospital database (Company: IQVIA, Frankfurt am Main, Germany), which contained data from 36 hospitals across Germany, including specialized hospitals, primary care hospitals, maximum care, standard care, and university hospitals. The dataset is a standardized data format transmitted by hospitals to the Reimbursement Institute for Hospitals (InEK) following §21 of the Hospital Compensation Act (KHEntgG). The individual treatment episodes included in the §21 dataset of a case were grouped using special grouper software developed by 3M Health Information Systems and IQVIA. In addition, the export files generated by the software were anonymized (e.g., case and patient number) for data protection reasons before transmission.

### 2.2. Study Population

The inclusion criteria for the present study were (1) hospital admission between January 2019 and December 2023, (2) a primary diagnosis of AMI (ICD-10: I21), and (3) age ≥ 18 years. No specific exclusion criteria were defined.

### 2.3. Study Outcome and Covariables

The outcome of the study was the prevalence of in-hospital mortality as a function of sex. The dataset contains death as one of the discharge types. The proportion of deceased patients was calculated separately for women and men, as well as being stratified by age group (<50, 50–59, 60–69, 70–79, >80 years), as well as AMI type, including ST elevation (STE-MI) myocardial infarction (ICD-10: I21.0, I21.1, I21.2, I21.3) and non-ST elevation (NSTE-MI) myocardial infarction (ICD-10: I21.4).

To describe the study population, along with age and MI type, secondary diagnoses found in at least 5% of the study population were given. These diagnoses included hypertension [ICD-10: I10], hypothyroidism [ICD-10: E03], type 2 diabetes mellitus [ICD-10: E11], lipid metabolism disorders [ICD-10: E78), chronic ischemic heart disease [ICD-10: I25], hypokalemia [ICD-10: E87.6], atrial fibrillation and flutter [ICD-10: I48], heart failure (ICD-10: I50), nonrheumatic valve disorders (ICD-10: I34-I37), atherosclerosis (ICD-10: I70), chronic obstructive pulmonary disease (COPD, ICD-10: J44) and chronic kidney disease (ICD-10: N18).

### 2.4. Statistical Analyses

Differences in the sample characteristics and secondary diagnosis prevalence of the secondary diagnosis between female and male patients were compared using the T-test for age and the Chi^2^ test for categorical variables. To assess the associations between female sex vs. male sex with regard to in-hospital mortality, multivariable logistic regression analyses were conducted, adjusted for age, MI type, and secondary co-diagnoses. Models were conducted for the total population and by age group and MI type. The results of the logistic regression models were given as the odds ratio (OR) with 95% confidence intervals (CIs). Due to multiple comparisons, *p*-values < 0.01 were considered statistically significant only. All analyses were performed using SAS 9.4 (SAS Institute, Cary, NC, USA).

## 3. Results

### 3.1. Baseline Characteristics

The present study included 9142 male and 4128 female patients with acute MI. Women were significantly older than men (74.4 years versus 67.7 years). The proportion of NSTE-MI was higher in women than in men (70.7% versus 66.7%). Chronic ischemic heart disease (91.6% versus 81.5%), atrial fibrillation and flutter (23.4% versus 19.6%), nonrheumatic valve disorders (11.3% versus 7.3%), hypothyroidism (18.8% versus 6.6%), hypokalemia (13.8% versus 9.5%), and chronic kidney disease (17.6% versus 12.6%) were more frequent in women, while lipid metabolism disorders (52.2% versus 45.2%) were more frequent in men (Table 1).

### 3.2. Prevalence of In-Hospital Mortality

**Figure 1:**
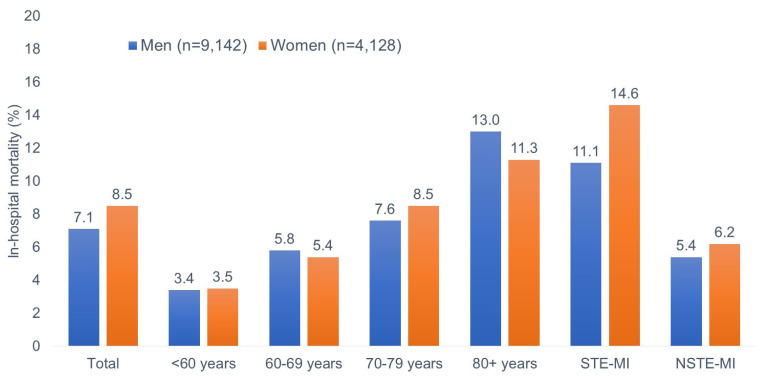
In-hospital mortality in patients with acute myocardial infarction by sex.

Figure 1 shows the proportion of fatal outcomes by sex. Overall, in-hospital mortality was higher in women than in men (8.5% versus 7.1%). However, the difference between in-hospital mortality between women and men varied by age. In the 70–79 age group, women had a higher mortality rate (8.5% versus 7.6%) whereas in the elderly group (>80 years), the proportion of deaths was higher in men compared to females (13.0% versus 11.3%). In the MI-type stratified analyses, the in-hospital mortality rate was higher in women in both MI types; however, in STE-MI (14.6% of women versus 11.1% of men), the difference was stronger than in NSTE-MI (6.2% of women versus 5.4% of men) (Figure 1).

### 3.3. Association Between Female Sex and In-Hospital Mortality

In a multivariable regression model, female sex was not significantly associated with in-hospital mortality (OR: 0.89; 95% CI: 0.77–1.04) irrespective of the MI type. In the >80 age group, female sex was associated with a lower in-hospital mortality rate (Table 2).

## 4. Discussion

The main finding of the present study is the lack of significant association between female sex and in-hospital mortality irrespective of age in Germany. This finding aligns with some other German [8] and American studies [4] that demonstrated no significant sex-based differences in in-hospital mortality among AMI patients. In contrast, other data from the USA and Spain suggested that women had significantly higher in-hospital mortality than men [7,11] and female sex had a negative impact on most of the outcomes in younger (<45 years) and middle age (45 to 64 years) [7]. These discrepancies could be due to outdated data (2003–2015, 2005–2015) but also methodological and healthcare differences.

The higher rate of in-hospital mortality among women in other countries may be due to comparatively lower chances of receiving invasive treatment across all age groups [7] or excessive delays (e.g., in-hospital patient delays, pre-hospital system delays, and hospital delays) [12]. These aspects in turn may be explained at least in part by differences in symptom presentation. Women more often experience atypical or less recognizable symptoms, leading to delays in diagnosis (first medical contact and door-to-balloon time) [5] and treatment [13] or altered response to treatment. These facts somewhat explain the increased risk of death [14,15] and the higher 30-day mortality among women compared to men [16].

However, our findings suggest that women in the German healthcare context may not experience the same in-hospital mortality disadvantage seen in other countries—possibly due to equitable care, standardized protocols, and early intervention. First, Germany has a well-structured, accessible healthcare system with near-universal insurance coverage. This may reduce disparities in treatment delays and quality of care [17]. Second, the presence of nationwide protocols and quality control programs (e.g., through German Cardiac Society initiatives) may ensure that both men and women receive guideline-based treatments at similar rates, which can attenuate mortality differences [18]. Third, physicians in the hospitals included in the present study may have been more sensitized to atypical AMI presentations in women due to ongoing education and training, reducing the diagnostic delays that disproportionately affect women in other settings.

On the other hand, this study is subject to a number of limitations that should be noted when interpreting the findings. First, our study was a retrospective study, meaning that we were unable to correct for all potential confounders; only the variables available were adjusted during the analysis. The database used did not include data on socioeconomic factors (such as education and income), lifestyle-related risk factors (such as smoking, alcohol consumption, or physical activity), or medication therapy such as antiplatelet drugs, anticoagulants, or antihypertensives. Second, patients who were transferred to hospitals were not included in the study, potentially leading to an underestimation of mortality, particularly in women.

## 5. Conclusions

In the present study, no significant sex-based differences in in-hospital mortality among patients suffering from AMI in Germany were observed. There is a need for further investigation regarding the impact of sex on AMI mortality with the help of longitudinal studies in different populations and geographies.

## Figures and Tables

**Table 1:** Baseline characteristics of the study sample after 1:1 matching.

	Women (n = 4128)	Men (n = 9142)	*p*-Value
Age			
Mean age (SD)	74.4 (12.3)	67.7 (12.8)	<0.001
<60 years, n (%)	651 (13.6)	2583 (28.3)	<0.001
60–69 years, n (%)	729 (17.7)	2401 (26.3)	
70–79 years, n (%)	1088 (26.3)	2158 (23.6)	
80+ years, n (%)	1750 (42.4)	2000 (21.9)	
MI type, n (%)			
STE-MI	1208 (29.3)	3041 (33.3)	<0.001
NSTE-MI	2920 (70.7)	6101 (66.7)	
Secondary diagnosis, n (%)			
Hypothyroidism	774 (18.8)	607 (6.6)	<0.001
Diabetes mellitus	1168 (28.3)	2573 (28.1)	0.859
Lipid metabolism disorders	1865 (45.2)	4773 (52.2)	<0.001
Hypokalemia	570 (13.8)	871 (9.5)	<0.001
Hypertension	2567 (62.2)	5622 (61.5)	0.450
Chronic ischemic heart disease	3366 (81.5)	8374 (91.6)	<0.001
Atrial fibrillation and flutter	967 (23.4)	1795 (19.6)	<0.001
Heart failure	1336 (32.4)	2778 (30.4)	0.023
Nonrheumatic valve disorders	467 (11.3)	670 (7.3)	<0.001
Atherosclerosis	224 (5.4)	469 (5.1)	0.478
COPD	282 (6.8)	484 (5.3)	<0.001
Chronic kidney disease	728 (17.6)	1151 (12.6)	<0.001

**Table 2:** Association of female sex with in-hospital-mortality in patients hospitalized for myocardial infarction in different subgroups (multivariable logistic regression).

	Multivariable Model	
Subgroup	OR for Females Versus Males (95% CIs)	*p*-Value
Total	0.89 (0.77–1.04)	0.131
<60 years	1.10 (0.64–1.89)	0.739
60–69 years	0.90 (061–1.33)	0.590
70–79 years	1.02 (0.76–1.36)	0.896
80+ years	0.79 (0.64–0.97)	0.027
STE-MI	0.99 (0.78–1.25)	0.910
NSTE-MI	0.84 (0.69–1.03)	0.094

## Data Availability

The datasets used and analyzed during the current study are available from the corresponding author upon reasonable request.
